# Cross-Layer Service Discovery Mechanism for OLSRv2 Mobile *Ad Hoc* Networks

**DOI:** 10.3390/s150717621

**Published:** 2015-07-20

**Authors:** M. Isabel Vara, Celeste Campo

**Affiliations:** Department of Telematic Engineering, University Carlos III of Madrid, Avda. de la Universidad 30, 28911 Leganes, Madrid, Spain; E-Mail: maribelvaralorenzo@gmail.com

**Keywords:** service discovery, OLSRv2, MANET

## Abstract

Service discovery plays an important role in mobile *ad hoc* networks (MANETs). The lack of central infrastructure, limited resources and high mobility make service discovery a challenging issue for this kind of network. This article proposes a new service discovery mechanism for discovering and advertising services integrated into the Optimized Link State Routing Protocol Version 2 (OLSRv2). In previous studies, we demonstrated the validity of a similar service discovery mechanism integrated into the previous version of OLSR (OLSRv1). In order to advertise services, we have added a new type-length-value structure (TLV) to the OLSRv2 protocol, called service discovery message (SDM), according to the Generalized MANET Packet/Message Format defined in Request For Comments (RFC) 5444. Each node in the *ad hoc* network only advertises its own services. The advertisement frequency is a user-configurable parameter, so that it can be modified depending on the user requirements. Each node maintains two service tables, one to store information about its own services and another one to store information about the services it discovers in the network. We present simulation results, that compare our service discovery integrated into OLSRv2 with the one defined for OLSRv1 and with the integration of service discovery in *Ad hoc* On-demand Distance Vector (AODV) protocol, in terms of service discovery ratio, service latency and network overhead.

## 1. Introduction

Mobile *ad hoc* networks are one of the emerging trends in wireless communication thanks to their wide application field. It is now common to find personal electronic devices, such as smartphones and wearables computers (smart-watches, smart-glasses, *etc.*) with communication capabilities based on WiFi and/or IEEE 802.15 technologies that allow users to communicate and share information with each other in a network with a minimal deployment and without the need for an underlying infrastructure. Today, for example, it is possible for users attending a conference to share presentations, print documents or access the Internet without any configuration on their computers. At home, it is possible to adjust remotely the lighting and temperature or to change the music. On the road, vehicles can cooperate and deliver information about the state of the road. Military, weather prediction, agriculture, natural disasters areas, search and rescue operations and any other situation where infrastructure is either absent or unavailable are other examples of different kinds of *ad hoc* networks.

Mobile *ad hoc* networks consist of a collection of wireless mobile nodes that dynamically exchange data among themselves without the reliance on a fixed base station or a wired backbone network. These nodes are free to move, to join or to leave the network at any time. They move randomly and organize themselves on a random basis. This high mobility of nodes causes the network topology to change rapidly and unpredictably. Moreover, these nodes may run out of battery power, fail or decide to shut down at any time. Thus, a MANET is a self-created, self-organized and self-administrated network. It is a network with neither fixed infrastructure requirements nor centralized management for its operation. The network nodes work together with minimal central control and human intervention. All of the nodes of these networks behave as routers and take part in discovery and maintenance of routes to other nodes in the network. Nodes in the network cooperate in routing and forwarding. Most of them have limited computational capabilities, battery life, memory availability and CPU performance capacity.

When the *ad hoc* network is established, nodes will run different applications. These applications will usually provide and request services in the *ad hoc* network. In these terms, service discovery is an important area. Service discovery architectures let the nodes discover services, as well as to use the available services in the network. The automatic discovery of services is therefore a crucial feature in mobile *ad hoc* networks. Systems and protocols that provide service discovery solutions are plentiful and diverse, although most of them are not appropriate for mobile *ad hoc* networks. Traditional service discovery strategies are defined for an almost completely static environment with rare network topology changes. The architecture of these protocols is centralized, with servers storing services in a central node directory, not designed for networks with mobile nodes. Service discovery protocols designed for *ad hoc* networks require a distributed approach where server nodes should also be autonomous and able to announce their presence and the services that they provide.

The problem of service discovery was first considered for the Internet, with the creation of the Internet Engineering Task Force's (IETF) Service Location (SRVLOC) group in 1993. The main result of the group was the definition of the Service Location Protocol (SLP). SLP Version 2 is currently an Internet standard (Request For Comments (RFC) 2608) [1]. This protocol was defined at the application layer, and then, the first service discovery protocols for MANETs have been also defined at this layer [2,3]. However, these protocols do not provide any information about the route to the service provider. In order to obtain this information, a separate routing process is required. Therefore, we need two separate processes: one to discover the server node that provides the service and a second one to obtain a route to that node. The effect of combining these two separate processes is known as “cross-layer service discovery”.

Integration of routing and service discovery is an idea that was first introduced by Koodli *et al.* [4]. Now, there are several different proposals that integrate service discovery in reactive, as well as in proactive routing MANETs. Cross-layer service discovery optimizes control overhead and reduces service acquisition latency [5]. For any node seeking a service, the route to the server node is known at the same time as the service provider.

The main focus of this article is to present a service discovery mechanism integrated into the OLSRv2 [6] protocol that supports service advertisements in *ad hoc* networks. This mechanism was previously defined for OLSRv1 [7] in [8]. In [9], we gave new results that demonstrate that the protocol could be a serious candidate for discovering services in proactive *ad hoc* networks. Since then, the mechanism has suffered some changes to integrate it into OLSRv2. We will explain these changes, and we will also give new results about the performance of the service mechanism for different *ad hoc* network scenarios. We compare the results obtained with the solution described for OLSRv1.

We also compare our new design to a service discovery solution over the *Ad hoc* On-demand Distance Vector (AODV) [10] reactive routing protocol. We evaluate the approaches thoroughly using simulations, and we conclude that the new design over OLSRv2 is superior to the one over OLSRv1. Moreover, in large and high mobility networks, the OLSRv2 service discovery solution behaves better than the AODV service discovery protocol, regarding service availability, service latency and network overhead. The improvements of the OLSRv2 protocol and the characteristics of proactive networks make the new design a serious candidate for service discovery in large and high mobility *ad hoc* networks.

The remainder of this paper is organized as follows: In Section 2, we present an overview of MANET routing protocols, and we briefly review OLSRv1 and OLSRv2 protocols. In Section 3, we discuss the need for a cross-layer approach for service discovery in *ad hoc* networks and the implications of integrating service discovery with routing. Section 4 gives a short overview of the research efforts made in service discovery in MANETs. Section 5 describes our solution for integrating service discovery information into the OLSRv2 routing protocol. Section 6 details the results from our simulation experiments. Finally, Section 7 concludes the paper and refers to our future research directions.

## 2. Routing Background

In a large MANET, intermediate nodes acting as routers are needed, so that a node is able to communicate with a distant node out of its coverage range. Two things are required in order to make this multihop routing work: firstly, the network must be dense enough to guarantee that at least there is one path between the source and destination [11,12]; secondly, a routing protocol is needed to find this path.

Routing protocols for MANETs are different from routing protocols for fixed networks. In fixed networks, only a few nodes act as routers, but in MANETs, almost all of the nodes have to act as routers to maintain network connectivity among all of the nodes. Besides, due to the highly dynamic nature of MANETs, the low capacity of wireless links, the limited bandwidth and the power constraints of mobile nodes, the routing strategies designed for wired networks are not suitable for a mobile *ad hoc* environment.

Two main different routing approaches are considered in mobile *ad hoc* networks: (1) table-driven protocols or proactive protocols; and (2) on-demand protocols or reactive protocols.

Proactive protocols are those where nodes continuously search for routing information within a network. Therefore, when a route is needed, it is already known. Nodes always have an updated table to know which path to use towards a destination. They offer fast response times and are adequate for real-time scenarios. Many proactive routing protocols have been proposed. The most representative one is the OLSR protocol.

On the other side, reactive protocols do not take the initiative to find a route to the destination until a node requires it. This kind of protocol only attempts to discover routes on demand, when a node needs to send data to an unknown destination. Thus, a periodic update of topology information is avoided. This way, reactive routing protocols reduce control data overhead and consumed bandwidth in the network at the cost of increasing the route acquisition latency to find routes to the destination. AODV is the most representative routing protocol for reactive protocols.

### 2.1. OLSRv1 and OLSRv2 Routing Protocols

OLSRv1 is the first version of OLSR defined in RFC 3626. It was developed by the French National Institute for Research in Computer Science and Control (INRIA) for MANETs. In OLSRv1, each node maintains topology information by periodically exchanging link-state messages. This characteristic lets OLSR have reliable routes to all nodes in the network when required. To adapt itself to the low bandwidth requirements of *ad hoc* networks, OLSR uses multipoint relays (MPR). Using the MPR technique, OLSR reduces the control traffic overhead and minimizes flooding. These optimizations make OLSRv1 suitable for use in large and dense networks, as the MPR optimization is especially effective in such situations.

HELLO messages are exchanged between neighbor nodes to obtain neighborhood information. By using this neighborhood information, each node selects a subset of one-hop away neighbors known as the MPR nodes set. In the MPR set, all two-hop away neighbors are reachable through any member of the MPR set. MPR nodes are the only ones to forward topology control (TC) messages periodically. The purpose of TC messages is to broadcast link state information and to transmit topology information to the entire network.

OLSRv2 is the second version of the OLSR protocol defined in RFC 7181. OLSRv2 holds the key features of OLSRv1, such as MPRs, and it includes some improvements on the protocol, such as a more modular and flexible architecture.

While in OLSRv1, HELLO and TC messages share a common header format, OLSRv2 uses the Generalized MANET Packet/Message Format defined in RFC 5444 [13], type-length-values (TLVs) specified in RFC 5497 [14] and, optionally, message jitter specified in RFC 5148 [15].

Generalized MANET Packet/Message Format specifies the syntax of a packet that is able to carry multiple messages required by a MANET routing protocol. Each message consists of a message header to identify the message type and a message body.

The OLSRv2 message header allows nodes to decide whether to process and/or to forward messages. The message body contains compressed addresses. If required, the message body also contains attributes. It is important to note that TLVs are used to represent these attributes.

**Figure 1:**
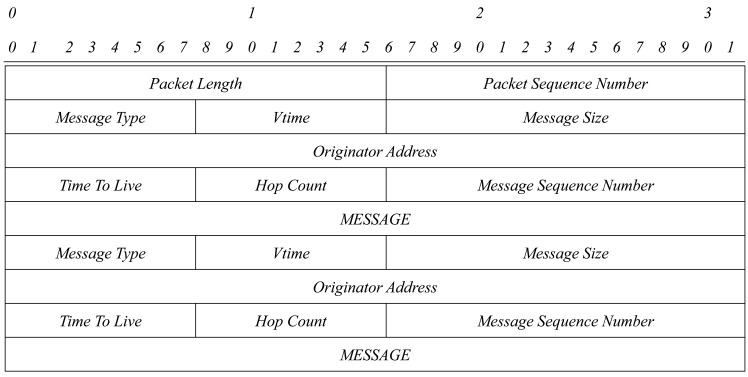
OLSRv1 packet format.

**Figure 2:**
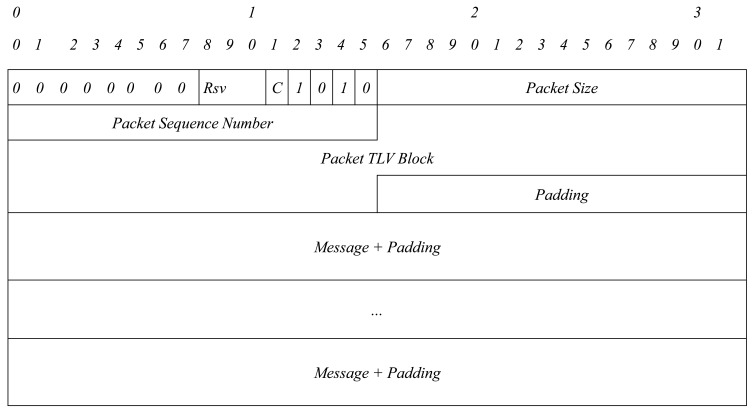
MANET packet format.

**Figure 3:**
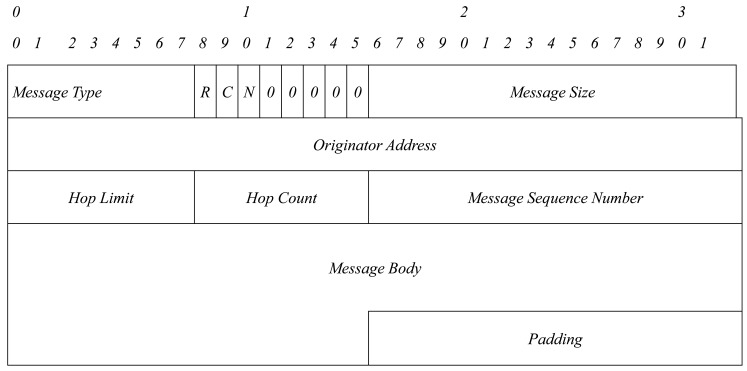
MANET message format.

In Figures 1–3, an OLSRv1 packet format and Generalized MANET Packet and Message Formats used in OLSRv2 are shown.

OLSRv1 uses HELLO messages to obtain neighborhood information, and OLSRv2 uses the Neighborhood Discovery Protocol (NHDP) [16] for neighbor discovery.

OLSRv2 also differs from OLSRv1 because it uses a link metric instead of hop count, as the OLSRv1 does, in the selection of shortest routes. Minimum hop routes are not often the best, for example three good links may be better than two poor links.

Finally, OLSRv2 includes extensions for security. While OLSRv2 allows signatures to be attached as packet/message TLVs, OLSRv1's immutable packet/message format causes signature problems.

## 3. Cross-Layer Design for Mobile *Ad Hoc* Networks

The characteristics of wired networks allow us to design a TCP/IP protocol stack with strict boundaries and to design protocols at different layers independently. However, this design is not flexible enough to cope with the characteristics and restrictions of MANETs [17,18].

Cross-layering means the interaction of a layer with any other layer in the protocol stack. The *ad hoc* research community recognizes that cross-layering can provide significant performance benefits. A cross-layer design may eliminate redundant tasks found on adjacent layers and benefit from the stack wide layer interdependencies to optimize overall network performance. Recent studies reveal that cross-layer integration result in significant improvement in terms of energy efficiency [19,20].

A cross-layer design applied to service discovery refers to service discovery solutions integrated into a routing protocol that disseminate service discovery and route discovery messages throughout the network at the same time. Traditionally, service discovery is placed at the application layer, arguing that cross-layer solutions violate a modular layered approach. An application layer service discovery solution means that clients must first discover the server that provides the service they desire, and then, once clients know the address of the server, they must initiate a separate routing process in order to obtain a route to the service provider. This means that two separate processes are required for any client intending to access a service, a service discovery and a route discovery process. Both of them would require the exchange of a large number of messages. In an *ad hoc* network, each message sent through the network consumes significant network bandwidth, as well as computation and battery power. Cross-layer service discovery solutions reduce significantly the amount of transmitted messages and the consumed bandwidth in the network [21–23]. A node requesting a service discovers the service and the route to the service provider at the same time. Cross-layer service discovery solutions also reduce the time to access a server, since the discovery of the server and the route to reach the server are obtained at the same time and not sequentially, as in the application layer service discovery approach.

## 4. Related Research on Service Discovery Protocols

Traditionally, service discovery architectures are classified into two types: directory-based architectures and directory-less architectures. Surveys that focus on service discovery in both fixed and *ad hoc* networks can be found in [24,25].

### 4.1. Directory-Based Architecture

The directory-based architecture can be also divided into two categories: centralized directory architecture and distributed directory-based architecture. A centralized directory architecture relies on central directories to store services of node providers. Most of the existing service discovery protocols based on a directory architecture are designed for fixed and infrastructure-based networks, and thus, they are neither efficient, nor practical for mobile *ad hoc* applications. SLP, Jini [26], Universal Plug and Play (UPnP) [27], Salutation [28] and Service Discovery Protocol (SDP) [29] are some examples of such protocols.

In MANETs, however, the network topology is very dynamic, and nodes have limited energy and bandwidth resources. It is not possible to statically configure nodes with a list of central directories or servers. Taking into account these characteristics, we hold that distributed directory architectures are more appropriate for MANETs than centralized directory architectures.

Recently, a considerable amount of work has been done on this research area. There are solutions that offer a cluster of service providers to be a service directory for answering service queries and accepting service registrations. Buvana *et al.* [30] propose a model for energy efficiency and scalability. Service discovery messages are flooded over the cluster head nodes, which form a service repository for service registration. Monire [31] proposes a solution where nearby nodes are grouped together to form clusters. Clusters are two hops in diameter, and they are overlapping or disjoint. Each cluster chooses a cluster head that coordinates the cluster members. Inter-cluster routes are dynamically discovered. Each node maintains a neighbor table and a cluster adjacency table. Siddarth *et al.* [32] propose a service discovery architecture using swarm intelligence. Swarm intelligence is used to establish the intra- and inter-cluster shortest path routing. Clients send a service request with its required quality of service (QoS) parameters to its source cluster head. Each cluster head searches the QoS-aware server with matching QoS constraints by means of a service table and a server table. Jayapal *et al.* [33] present an adaptive service discovery protocol that enhances the performance of service discovery. They use a distributed directory-based service discovery mechanism that operates in a proactive mode with service advertisements to the core node and selects a provider based both on the distance and service capability of the provider.

### 4.2. Directory-Less Architecture

Due to MANETs' characteristics, a directory-less architecture is more appropriate than a directory-based architecture. There are no directory agents in a directory-less architecture. There are only user and service agents. User agents send service queries when they need to discover a service, and service agents announce service advertisements periodically in order to achieve a high service availability.

In recent years, various protocols have been proposed. Oikonomou *et al.* [34] present a probabilistic, hybrid, directory-less service discovery mechanism called ADDER. ADDER has been designed for military IPv6-based MANETs. It achieves very low service acquisition time through the exchange of a very small number of short messages. The propagation of service descriptions is based on a distance vector algorithm. Campo *et al.* [35] define the Pervasive Discovery Protocol (PDP), a fully-distributed protocol that merges characteristics of both pull and push solutions. Devices maintain a list of services previously announced by others. For each advertisement, an availability time is included. PDP assumes that the underlying network is either a one-hop network or a multi-hop network with multicast network support. Kniess *et al.* [36] propose a service discovery protocol that applies a data aggregation scheme in intermediate nodes to reduce messages replies in MANET networks. Chakraborty *et al.* [37] propose a Group-based Service Discovery protocol (GSD) for pervasive environments. Nodes in GSD send periodic advertisements of services. The broadcast is limited not to consume a lot of bandwidth. GSD does not describe how a route to a server node is obtained. Neogy *et al.* [38] present a novel service discovery protocol using mobile agents that select their route dynamically and exchange service information with the nodes in order to speed up the process. The smooth random mobility model (SRMM) is used to estimate node location at a particular time. Weng *et al.* [39] present an adaptive directory-less service discovery system for MANETs that couples to the underlying geographic routing algorithm and sets configurable service renewal. There are other solutions where intermediate nodes answer service queries, such as [40], where Palmieri proposes a novel service discovery approach based on a fully-distributed and parallel search model that does not require any centralized intelligence, fixed roles and a stable communication infrastructure. Any node in the network that receives service announcements from service providers stores the services in order to answer service queries. This way, service queries are not forwarded up to the service providers.

#### 4.2.1. Service Discovery Integrated with Routing

Integration of service discovery with an underlying routing protocol was first proposed by Koodli *et al.* [4] and described in an Internet Draft. In their approach, service discovery requests (SREQ) are piggybacked on routing request packets (RREQ) and service discovery replays (SREP) on routing reply (RREP) messages. This solution relies on a reactive routing protocol, such as AODV. Now, several different proposals exist for reactively-routed MANETs, as well as for proactively-routed MANETs.

#### 4.2.2. Service Discovery in Reactive MANETs

Most of the service discovery architectures designed for reactively-routed MANETs rely on AODV as a routing protocol. In [22], the authors compare service discovery functionality integrated into AODV with a pure application-based service discovery protocol, called Nom. In [41,42], the authors added additional extensions to SD-AODV to support QoS-aware service selection. Service Discovery and Interaction with Routing protocols in Ad hoc Network (SEDIRAN) [43] and the proposal of Engelstad *et al.* [44] are other examples of such architectures. Recently, Bhumika *et al.* [45] extended the service discovery approach used in [22] in order to discover services within the network efficiently and to communicate with the server with the highest trust value. Zhong *et al.* [46] also proposed a new service discovery protocol called SCAODV, which integrates the route protocol and the process of service discovery.

#### 4.2.3. Service Discovery in Proactive MANET

Most of the service discovery architectures designed for proactively-routed MANETs rely on OLSR as a routing protocol. Due to its proactive nature, the OLSR protocol always has the routes to the nodes in the network immediately available when a node needs them. Integrating service discovery with this kind of protocol lets a node discover a service almost immediately. The main protocols defined so far are described below: the Mercury [47] service discovery protocol describes service information using Bloom filters. The protocol is fully distributed, and it utilizes intelligent local caching with service handover support. It introduces a new message called Mercury service discovery (MSD). This message contains a field to indicate whether the message is an advertisement or a query request, so that the nodes that compound the network can advertise, as well as discover services. Li *et al.* [48] proposed a service discovery scheme that discovers Session Initiation Protocol (SIP) servers in an *ad hoc* network. They define a new message type called service location extension (SLE) that is added to the OLSR routing protocol. SIP servers can periodically advertise their location using the SLE messages. Clients can also use SLE messages to request the location of a SIP server. García *et al.* [49] presented a distributed protocol for dynamic node IP assignment in MANETs. In this protocol, each node is responsible for managing a range of addresses. When a new node wants to begin participating in the network, one of the nodes within the network gives half of its address range to the new node. In the case of any adjacent node not having free addresses, but free addresses do exist, a request to a network node that has a free addresses is done. When a node leaves the network, it does not inform the server of its departure. Heni *et al.* [50] present a service discovery mechanism that integrates service discovery messages with OLSR TC messages.

## 5. Service Discovery Mechanism

In previous sections, we justified the use of a cross-layer approach to define an efficient service discovery protocol for MANETs. Based on this, in our previous works, we presented a new service discovery protocol based on OLSRv1. In 2014, a new version of OLSR was presented; then, we started to work on a new version of our service discovery protocol based on this second version of OLSR.

The behavior of the service discovery mechanism integrated into OLSRv2 is simple. Nodes that have one or more services to announce include service advertisements, as a TLV structure, in HELLO messages, with the name, a brief description of the service announced and a time to live parameter. HELLO messages are exchanged between neighbor nodes periodically, and all nodes at a one-hop distance receive them. Each neighboring node stores in a local remote service table the source node that offers the service, the service type, the time this service will be available in the network (time to live) and a brief service description.

At this point, it is worth mentioning that our protocol can use any kind of service description for service advertisements. In our implementation, we use a simple service description based on the format defined for the SLP protocol, but other more flexible service descriptions could be used to deploy our protocol in the Internet of Things (IoT) scenarios nowadays, so more advanced matchmaking techniques could be used to provide a better service discovery [51].

From all of the nodes that are at a one-hop distance, only MPR nodes retransmit service advertisements in the network. MPR nodes use TC messages to retransmit service announcements. For that purpose, TC messages also include a TLV structure with the same attributes as HELLO messages. This way, all of the nodes in the network are aware of the services announced within it.

When a node needs a service, it searches for servers on its remote service table. The route to reach a server is available through the routing table.

On the other hand, when a node leaves the network, it sends a notification message with the time to live parameter equal to zero. This means that the service or services that this node offers are no longer available.

### 5.1. SDM Message Format

In previous works [8,9], we showed the feasibility of this service discovery protocol, as a proactive push-based service discovery mechanism, integrated into the OLSRv1 protocol for MANET networks. We analyzed the service discovery mechanism in terms of messaging overhead, service discovery ratio (efficiency of the service discovery) and latency. The open lines were to extend the implementation of SDM messages and to integrate these messages into the new version of the OLSR protocol (v2), when it became an RFC standard (April 2014).

As we have seen, the OLSRv2 protocol offers several advantages over OLSRv1, so it is to be expected that our service discovery protocol probably would reach better performance results when we integrate it into OLSRv2: (1) OLSRv2 is a modular protocol (it complies with RFC 5444 to define packet and message formats); (2) OLSRv2's header package allows one to easily discriminate whether one received packet has to be processed or retransmitted; (3) messages and packets in OLSRv2 could be extended with attributes that follow a TLV structure; and (4) to find the shortest path to a destination, OLSRv2 does not use the lowest number of hops metric between source and destination, but the link state metric. Moreover, in experimental tests, OLSRv2 has demonstrated a better behavior than OLSRv1 [52]: (1) the throughput in OLSRv2 is significantly higher than in OLSRv1, understanding throughput as the number of packets sent with success per time unit; (2) the average end-to-end delay (mean time that a packet spends to reach its destination) is less in OLSRv2 than in OLSRv1; (3) the average jitter is 50% less in OLSRv2 than in OLSRv1; and (4) the battery consumption in the nodes at the end of a simulation experiment, in the same scenario, is lower in OLSRv2. This is a very important issue, because usually, a MANET is composed of battery-powered nodes.

To integrate the service discovery mechanism into OLSRv2, we define SDM messages as a TLV structure. In addition, we have made use of this new protocol design to improve our previous service discovery mechanism defined in OLSRv1: (1) the information in the routing tables is checked to know when a node leaves the network and so to delete the services offered by this node in the service table; and (2) we use the attributes VALIDITY_TIME and INTERVAL_TIME, already defined in RFC 5497, to configure the time to live parameter of a service in the network and the interval between service advertisements. We define INTERVAL_TIME as a user-configurable parameter, so depending on the working environment, this parameter could be higher or smaller.

**Figure 4:**
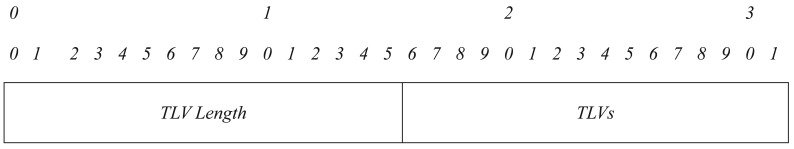
Type-length-value (TLV) structure format.

**Figure 5:**
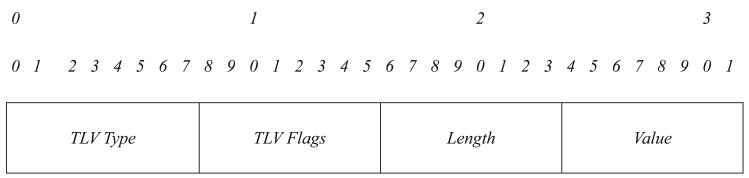
TLV block format.

Then, we just have to define two new TLV structures: SDM_TYPE and SDM_DESCRIPTION. Each TLV structure has the format shown in Figure 4. We group all TLVs into one TLV block. A TLV block format is shown in Figure 5.

**Figure 6:**
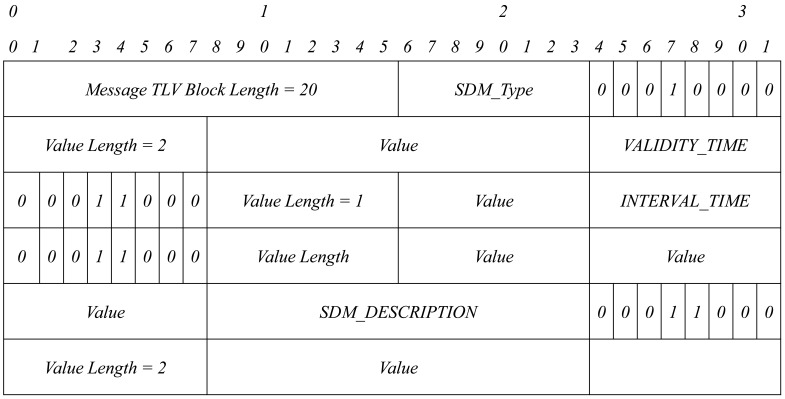
TLV block with four TLVs.

Figure 6 shows the format of a TLV block with the four TLV structures needed for service discovery.

**Figure 7:**
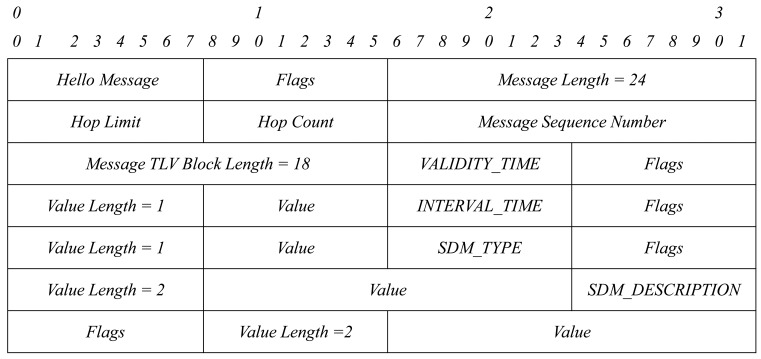
HELLO message format.

**Figure 8:**
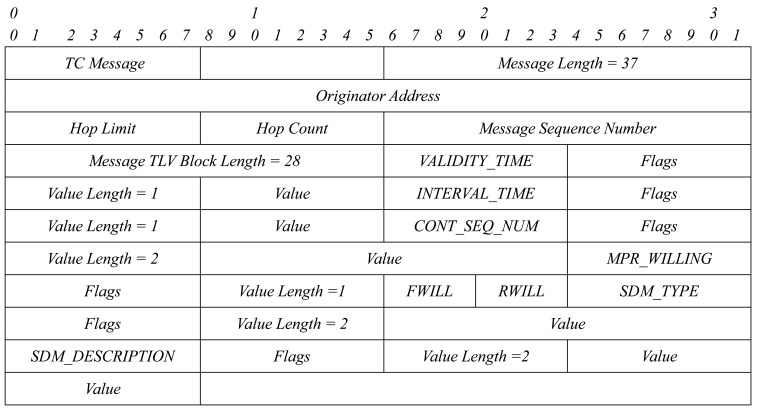
Topology control (TC) message format.

This TLV block is added to HELLO and TC messages when needed: in the event that a node has to announce a service and/or in the event that a node has to retransmit the service announcement of another node. The packet format will be as Figures 7 and 8 show.

### 5.2. SDM_TYPE TLV Generation

Services announced by each node are part of the HELLO and TC messages defined in the OLSRv2 protocol. Besides the TLV structures defined in OLSRv2, we have defined a new one for service discovery.

Taking into account the size of the OLSRv2 header and the size of the HELLO and TC messages, the size of an OLSRv2 packet with a TLV service discovery structure is the following:

OLSRv2 packet header + HELLO message + SDM_TYPE TLV = 34 bytes

OLSRv2 packet header + TC message + SDM_TYPE TLV = 39 bytes

In OLSRv1, when a HELLO and SDM message were transmitted, there was an overhead in the network of 54 bytes. In the case of TC messages, the number of bytes transmitted was 52.

HELLO messages with a SERVICE_DISCOVERY TLV structure are sent whenever: (1) a node has to announce a new service; (2) a node leaves the network, in this case the VALIDITY_TIME TLV structure will have a value of zero; and (3) periodically, when the timer in the INTERVAL_TIME TLV structure expires.

The shorter the time defined in the INTERVAL_TIME attribute is, the more accurate the services stored at the remote service table of each node are, but the bandwidth consumption in the network will be greater. On the contrary, the higher the time defined in the INTERVAL_TIME attribute is, the lower the bandwidth consumption is, but the nodes will not have a service table updated as desired. This implies that the false service discovery ratio will be increased. As INTERVAL_TIME is a user-configurable parameter, we modify it depending on the user requirements needed every moment.

### 5.3. SDM_TYPE TLV Forwarding

Only the nodes defined as MPRs are in charge of retransmitting messages with service announcements in the network. The service announcements are retransmitted with the attributes defined in a TLV structure as part of the TC messages.

### 5.4. SDM_TYPE TLV Processing and Service Table

**Figure 9:**
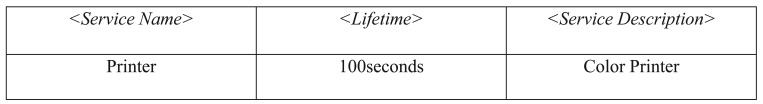
Local service table of each node.

Each node has a local service table where it stores its own services and another service table (remote service table) where it stores locally services announced by other nodes in the network. The local service table has the information shown in Figure 9.

**Figure 10:**
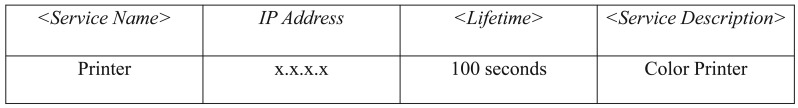
Remote service table of each node.

The *Service Name* parameter indicates the name of the service that will be announced; the *Lifetime* parameter indicates the time to live of the service in the network; and the *Service Description* gives a detailed description of the service. There will be as many entries in the local service table as services announced by this node. The remote service table of each node will be filled with the information received in HELLO and TC messages that have a TLV structure with service announcements. The remote service table of each node has the information shown in Figure 10.

It is possible for a node to receive a message with a service announcement that it already has in its remote service table. In this case, if the time to live of the service announced is greater than the time to live of the service stored in its remote service table, the node will update the information of the service table with the new values. In the case the service announced matches one of the services that this node offers, the service is not stored in its remote services table. Local services always prevail over remote services.

Whenever a node has a service to announce or a service update to send, it will use HELLO messages through the previously defined TLV structure. HELLO messages have a periodicity defined in the INTERVAL_TIME attribute. This attribute is a user-configurable parameter. When the *Lifetime* parameter of a service in the remote service table of each node reaches zero, the node waits the following INTERVAL_TIME to know if the service is no longer available in the network. If after that time, the service is not re-announced, the node deletes this entry from its remote service table. During this time, if a node wants to access the service, a false service discovery can happen.

### 5.5. Service Discovery

When a node wants to use a service, it first looks for that service in its local service table. If it is not there, it looks for the service in its remote service table in order to know if some other node in the network offers the service. If there is not an entry for this type of service in its remote service table, the service will not be available at this moment. Otherwise, if there is an entry for that service, we check if the node offering the service remains available, as explained below.

To guarantee that the node that offers the service remains available in the network and to decrease the false service discovery ratio, we check the information the node stores in the *Topology Information Base*. This information is updated with the information received in TC messages in the *Advertising Remote Router Set, Router Topology Set and Routable Address Topology Set* fields. Moreover, each node keeps a *Routing Set* updated with the information stored in *Topology Information Base, Local Information Base* and *Neighbor Information Base*.

The first set, *Advertising Remote Router Set*, stores information about what remote nodes transmit TC messages. Each entry has the following format: AR_orig_addr, AR_seq_number, AR_time. When the time defined in the AR_time parameter expires, the entry is deleted. In the *Router Topology Set*, information about link topology between MANET nodes is stored. Each entry has the following data: TR_from_orig_addr, TR_to_orig_addr, TR_seq_number, TR_metric, TR_time. TR_from_orig_addr is the node's address that can reach the node TR_to_orig_addr in one hop. In this case, the entry is deleted when the TR_time has finished. In the *Routable Address Topology Set*, topology information about routes in the network is stored; it contains data about through which nodes a destination is reachable: TA_from_orig_addr, TA_dest_addr, TA_seq_number, TA_metric, TA_time. TA_from_orig_addr is the node's source address that can reach the node TA_dest_addr in one hop. Furthermore, this entry is deleted when the TA_time has finished. Finally, the *Routing Set* stores information about the first hop of a path to each destination node reachable by this node: R_dest_addr, R_next_iface_addr, R_local_iface_addr, R_dist, R_metric. R_dest_addr is the destination address, and R_next_iface_addr is the network address of the “next hop” in the destination's path. R_local_iface_addr is the source address used to send the packet to the destination. R_dist is the number of hops to the destination. This information is updated (entries are added or deleted) with information obtained in *Link Sets, Neighbor Sets* and *Routable address Topology Set*.

At the same time, if an entry is deleted in these sets, this means that this node, or the route to it, is no longer available. In this case, we also delete the services offered by this node from its remote service table.

### 5.6. Service Unavailability

To decrease the false service discovery ratio, besides checking the topology information table, when a node leaves the network, it sends a notification message indicating that its services will not be available anymore. A TLV structure is also used for this notification. The node will announce the service SDM_TYPE, but with a time to live VALIDITY_TIME equal to zero. When the rest of the nodes receive this message, they delete these services from their remote service tables.

## 6. Simulation Environment and Results

### 6.1. Simulation Environment

The ns-2 simulation tool is used to evaluate the performance of the service discovery enhancements applied into OLSRv2 in a dynamic *ad hoc* environment. The ns-2 engine is written as a mixture of Tool Command Language (Tcl) and C++, and the underlying protocols are written almost completely in C++. Protolib [53] is a library that provides a set of simple, cross-platform C/C++ classes that allow the development of network protocols that can run on ns-2 as ns-2 agents.

We have used an OLSRv2 implementation in Java [54] and modified it to implement the improvements designed for service discovery.

We can run the JOLSR v2 implementation in ns-2 thanks to a tool named AgentJ [55] that allows us to use the Java routing protocol implementations within ns-2 as Java agents without modifying the source code of ns-2.

The presented experiments compare the new design of our service discovery protocol made for OLSRv2 with the previous one designed for OLSRv1. We also compare the performance of service discovery described here for the proactive OLSRv2 protocol with the service discovery for the reactive AODV routing protocol. We have modified the extension of AODV for service discovery proposed in [4] and implemented it here [56] to adapt it to our environment in ns-2.34.

In the simulation, each mobile node changes its location within the subnet based on the random waypoint model: the node randomly selects a destination and moves towards that destination, at a speed uniformly distributed between 0 m/s and some maximum speed. We limit the maximum speed of a node to 10 m/s. Once the node reaches its destination, it waits for a pause time before choosing a random destination and repeating the process. The pause time is set to 10 s. The mobility pattern is read from the mobility file. In this model, each node is placed randomly in the simulated area (1000 × 1000 m^2^). In ns-2, the distributed coordination function (DCF) of IEEE 802.11 for wireless LANs is used as the MAC layer protocol. The transmission range is about 250 m. The signal propagation model combines both a free space propagation model and a two-ray ground reflection model. See Table 1.

In the simulations, many different scenarios have been considered with different numbers of nodes, in order to simulate different density networks. These nodes enter the network at random times and also leave the network after a random time. Once in the network, each mobile node changes its position based on the random waypoint model. This means that the number of services in the network varies over time, but its mean remains stationary, because new users join the network at the same time rate that they leave it. Random times follow exponential distributions. For simplicity, we assume that only five nodes act as servers. Each server offers just one different service. We measure several significant metrics in order to evaluate the performance of the service discovery mechanism designed for OLSRv2 and its feasibility: (1) the overhead introduced to the network; (2) latency to find a service; (3) the service discovery ratio (the efficiency of the service discovery); and (4) the false service discovery ratio (services discovered that were not available in the network out of the total number of services discovered).

For the OLSRv1 and OLSRv2 parameters, we use a set of default parameters specified in both RFCs [6,7]. For AODV, a SREQ is generated every 5 s. Eighty service requests are made during the simulation interval. The values of service latency calculated are the average over all of the requests. We represent two different scenarios for AODV. In the first one, nodes broadcast their SREQs throughout the entire network. In the second one, the flooding is limited by a flooding scope parameter to two hops. This parameter defines the maximum number of hops a service discovery request is allowed to go in the network.

In order to gain good confidence in the obtained results, we run the simulations 10 times during 400 s with different mobility pattern files.

### 6.2. Simulations Results

#### 6.2.1. Network Overhead

First of all, we compare the service discovery mechanism over OLSRv2 with the one defined over OLSRv1, in terms of packet overhead in several scenarios, with several numbers of nodes: 10, 20, 30 and 50. In OLSRv1, we distinguished between HELLO, TC and SDM messages, because we wanted to know the overhead of SDM messages compared to HELLO and TC messages. In OLSRv2, we do not add any new messages, but we integrate the new TLV structure into HELLO and TC messages. Therefore, the comparison between both versions of the service discovery mechanism, will be based on the total number of transmitted packets. In terms of power consumption, minimizing the number of transmissions allows reducing battery consumption in limited nodes, although the packet size is greater [57].

We run simulations in three different scenarios. In each one, we modify the user-configurable parameter SDM INTERVAL_TIME to: (1) one second; (2) five seconds; and (3) ten seconds.

**Figure 11:**
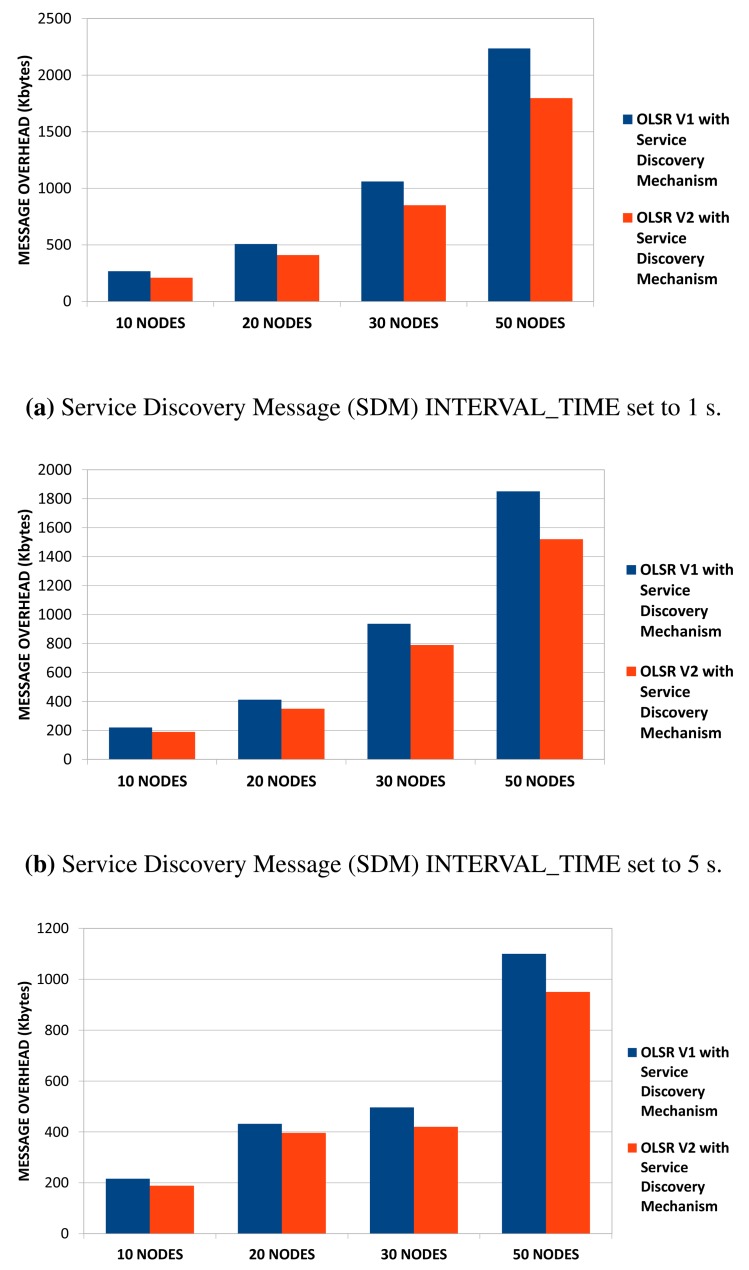
Network overhead in OLSRv1 and OLSRv2 with service discovery.

In Figure 11, we can see that OLSRv2 incurs less overhead in the network than OLSRv1 when services are announced. We also see that the difference is bigger as the number of nodes increases. Furthermore, the difference is greater as the announcement of services becomes less frequent. This result was expected because in OLSRv1, all of the HELLO, TC and SDM messages have the OLSR header, while in OLSRv2, the service announcement is integrated as a TLV structure in HELLO and TC messages. Anyway, for both OLSRv1 and OLSRv2, the smaller the SDM INTERVAL_TIME parameter is, the more overhead is introduced to the network. Although the overhead increment is more significant as the size of the network increases, in small networks, the SDM INTERVAL_TIME user-configurable parameter is not critical in terms of network overhead.

On the other hand, in order to compare network overhead introduced by service discovery in OLSRv2 and AODV, we use the total number of packets transmitted over the network. We vary node mobility from 2 m/s to 10 m/s in order to simulate networks with different mobility patterns. As we explained before, we simulate two different scenarios for AODV, one for broadcasting SREQs and the other one with a limited flooding scope for SREQs set to two hops.

**Figure 12:**
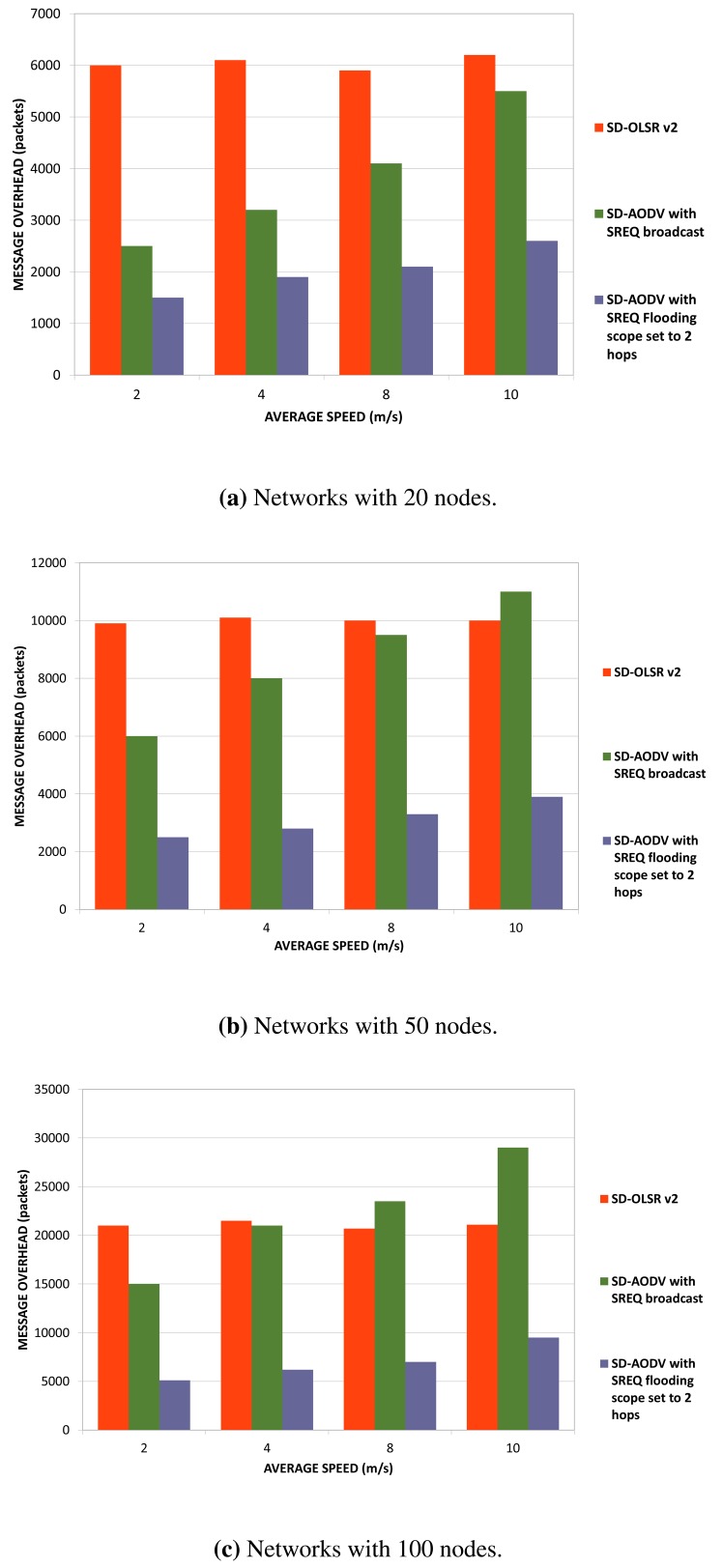
Network overhead in OLSRv2 and AODV with service discovery.

As we can observe from Figure 12, OLSRv2 service discovery (SD-OLSRv2) introduces more overhead than AODV service discovery (SD-AODV) in any of the scenarios simulated, except for the one without a flooding limited scope for SREQs and node average speed set to 10 m/s. OLSRv2 sends periodically route and service update information throughout the entire network. Although it uses MPRs to broadcast control messages within the network, this increases the network overhead. We also observe that in SD-OLSRv2, no additional overhead occurs as the network mobility becomes higher. The network overhead comes from periodic route and service updates and is independent of the traffic mobility patterns. On the contrary, SD-AODV introduces lower overhead than SD-OLSRv2 in networks with a low number of nodes with low mobility. As the size of the network increases and the nodes move faster, SD-AODV suffers more network overhead. If SREQs are broadcast throughout the entire network with node average speed set to 10 m/s, SD-AODV behaves worse than SD-OLSRv2 in terms of packet overhead. As we can observe in networks with 100 nodes, this fact becomes more significant as the size of the network increases.

#### 6.2.2. Service Discovery Delay

Now, we compare service discovery delay in OLSRv1 and OLSRv2 networks. We measure the service discovery delay according to the node speed in the network and the number of nodes offering the same type of service. By default, there are only five nodes announcing services. We do the comparison in simulation scenarios with 50 nodes. The parameters for sending periodic HELLO and TC messages are the ones established by OLSRv1 and OLSRv2 RFCs. SDM INTERVAL_TIME to announce services is set to 5 s.

**Figure 13:**
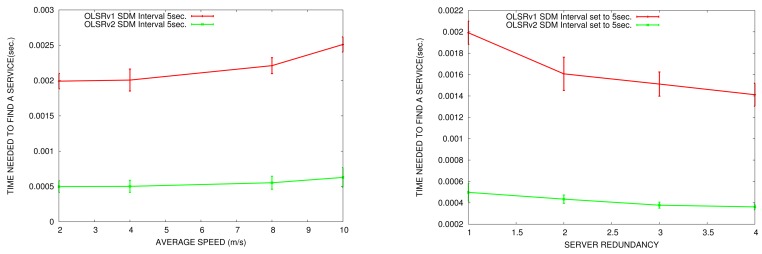
Service discovery delay *vs.* average speed (**left**) *vs.* service redundancy (**right**).

In Figure 13, we can see that in OLSRv2 networks, the delay to discover a service is lower than in OLSRv1 networks. This result is as expected, taking into account that the average end-to-end delay is less in OLSRv2 than OLSRv1 [52].

If we analyze the delay according to node speed, as the network changes more, the service discovery delay increases. When the network is more static, there is less probability of broken links and packet losses, so it is faster to find a service.

If we analyze the delay according to the number of servers, when the number of servers increases, the service discovery delay decreases, because there are more nodes that offer the same service.

**Figure 14:**
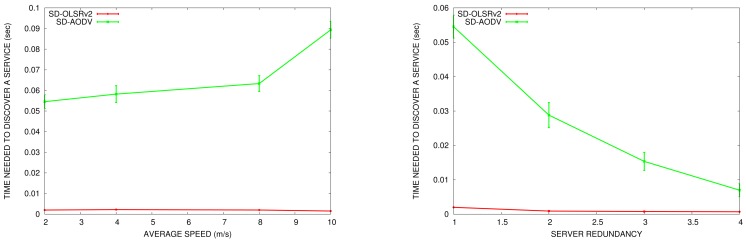
Service discovery delay *vs.* average speed (**left**) *vs.* service redundancy (**right**) in SD-OLSRv2 and SD-AODV.

On the other hand, in SD-AODV, the average time to access a service decreases as the server redundancy increases; Figure 14. However, whereas in SD-OLSRv2, services are available almost immediately, it takes hundredths of seconds in SD-AODV. This can be explained as follows. Proactive protocols produce higher routing efficiency than reactive protocols. OLSR maintains an up-to-date routing table at all times. When a node in a network running the OLSR protocol wants to find a route to a node, it only has to look in its routing table, whereas in an AODV network, a route discovery process has to be started. It goes without saying that looking in the routing table takes less time than flooding the network for a route discovery.

We also note that for SD-AODV, the average time to access a service increases in conditions of high mobility. In conditions of high mobility, a big number of routes will break, discovered routes may be invalid after a short period of time and, hence, a new route discovery process is needed. In SD-OLSRv2 services are available almost immediately, and it takes hundredths of seconds in SD-AODV.

#### 6.2.3. Service Discovery Ratio

In this section, we compare the service discovery ratio in OLSRv1 and in OLSRv2 according to the node speed and the number of nodes offering the same service in the network. We do the comparison in simulation scenarios with 50 nodes. The parameters for sending periodic HELLO and TC messages are the ones established by OLSRv1 and OLSRv2 RFCs. SDM INTERVAL_TIME to announce services is set to 5 s.

**Figure 15:**
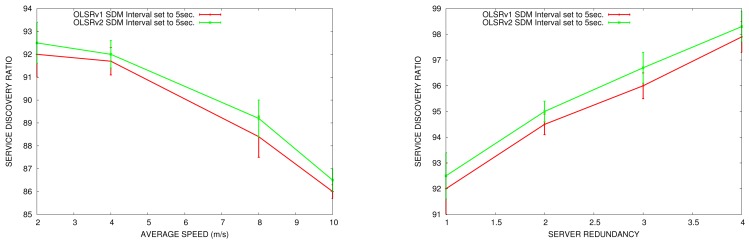
Service discovery ratio *vs.* average speed (**left**) *vs.* service redundancy (**right**).

In Figure 15, we can see that the service discovery ratio is slightly better in OLSRv2 than in OLSRv1. The basis for the service discovery mechanism is the same in both protocols, and this improvement is due to the advantages of the OLSRv2 itself, as it has better average end-to-end delay and throughput than OLSRv1.

Like the results obtained for service discovery delay, when the network is more mobile, the service discovery ratio decreases. Furthermore, when the number of servers increases, the service discovery ratio increases.

**Figure 16:**
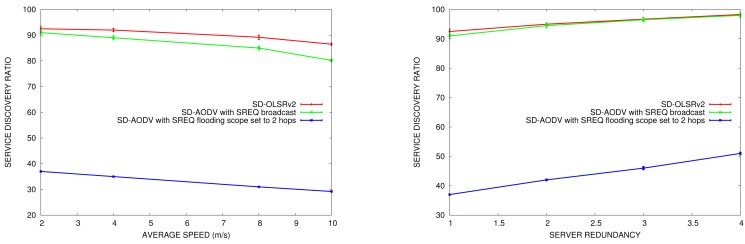
Service discovery ratio *vs.* average speed (**left**) *vs.* service redundancy (**right**) in SD-OLSRv2 and SD-AODV.

The comparison with SD-AODV gives us the results shown in Figure 16. For both SD-OLSRv2 and SD-AODV without an SREQ limited scope, the service discovery ratio is almost the same. If we limit the SREQ flooding scope, the service discovery ratio decreases significantly.

#### 6.2.4. False Service Discovery Ratio

In this section, we compare the false service discovery ratio in OLSRv1 and in OLSRv2 according to the node speed and the number of nodes offering the same service in the network. We do the comparison in simulation scenarios with 50 nodes. The parameters for sending periodic HELLO and TC messages are the ones established by OLSRv1 and OLSRv2 RFCs. SDM INTERVAL_TIME to announce services is set to 5 s.

**Figure 17:**
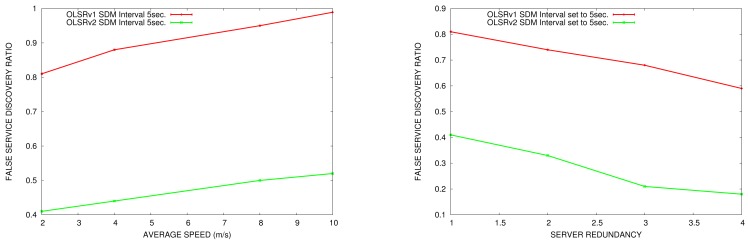
False discovery ratio *vs.* average speed ( **left**) *vs.* service redundancy (**right**).

In Figure 17, we can see that the false service discovery ratio is reduced in OLSRv2, since in OLSRv2, we check in the routing table if the node that offers the service is still in the network.

Like the results obtained for service discovery delay, when the network is more mobile, the false service discovery ratio increases. Furthermore, when the number of servers increases, the service discovery ratio decreases.

Regarding the false service discovery ratio in SD-AODV, with the scenario simulated in Table 1, the results obtained are similar to the ones in SD-OLSRv2, independent of the flooding SREQ scope. In SD-AODV, when a node needs a service for the first time, it requests it. If a service discover reply is obtained, there is no possibility of a false positive reply. However, when a node request a service repeatedly, it first verifies if it does not already have a route to the service provider. If it does, it does not request it. However, it may have the route active, when, actually, it is not. A false service discovery will occur.

## 7. Conclusions and Future Works

Service discovery is one of the most important mechanisms in an *ad hoc* network. Thanks to service discovery, service providers can announce their services in a dynamic way, and clients can discover and access those services in an autonomous manner.

The aim of this paper was to define a service discovery solution based on the one described for OLSRv1, but for the current version of the OLSR protocol. We have added a new TLV structure to the OLSR v2 protocol for service advertisements. We have also introduced some improvements to the service discovery mechanism, such as checking the *Topology Information Base* of each node to decrease the false service discovery ratio. One important part of the work was to evaluate by simulation whether the new version behaved better than the previous one in terms of overhead, time needed to find a service, service discoveries and false service discovery ratio when the mobility and the services of the network increased.

The simulation results showed that SDM over OLSRv2 is superior to SDM over OLSRv1 regarding the parameters described above. The service discovery delay decreases; the service discovery ratio is better; and the false service discovery ratio is reduced. Then, the improvements of OLSRv2 as a routing protocol also have a major impact on the performance of the service discovery protocol.

We also compared the performance of service discovery with AODV reactive routing protocol. We found that integrating service discovery with OLSRv2 gives better results in terms of service route acquisition latency regardless of the network size. In terms of service discovery, we do not find too many differences between both protocols, except when we limit service discovery request flooding scope in AODV. However, in terms of network overhead, SD-AODV gives better results than SD-OLSRv2, except when service discovery requests are broadcast throughout the entire network. SD-AODV gives better results at the expense of increased service acquisition latency. Overall, we conclude that integration of service discovery with OLSRv2 gives better results than integration with AODV for large and high mobility networks and, mainly, for networks where real-time services are desired. This will become more significant as the size of the network increases, due to proactive networks' characteristics.

For the future, we plan to test the proposed solution with larger networks. In OLSR, the denser and larger a network is, the more optimization is achieved. Therefore, it is expected to get better results in larger networks compared to the ones shown here.

We want to work on a real implementation of our protocol, including a new service description and advanced matchmaking algorithms to deal with the proliferation of new complex services in IoT environments. For reaching this objective, we will take into account the works on the Semantic Web of Things (SWoT) [51] of Information Systems Laboratory (SisInfLab), Information Systems Research Group (http://sisinflab.poliba.it).

On the other hand, we want to add additional extensions to service discovery integrated into OLSRv2, to support QoS-aware service selection. The goal of QoS-aware service selection is to select the node that optimize some QoS attributes, among other nodes offering equivalent services.

Finally, security issues are one of the open lines we are working on. MANETs are finding a place in real-world deployments, and it is fundamental to preserve the network connectivity from possible vulnerabilities and attacks. Along these lines, we are researching how nodes can select the most efficient security services in OLSRv2 with the minimum power consumption, the lowest packet overhead and the highest possible security level associated with the communications.

## Figures and Tables

**Table 1:** Simulation parameters. AODV, *Ad hoc* On-demand Distance Vector (AODV); SD, service discovery; OLSR, Optimized Link State Routing Protocol; DCF, distributed coordination function.

**Network Parameters**	**Value**
ns-2 Version	2.34
AODV Implementation	SD-AODV
OLSRv1 Implementation	UM(University of Murcia)-OLSR
OLSRv1 Parameters	Default values
OLSRv2 Implementation	JOLSRv2
OLSRv2 Parameters	Default values
Simulation Time	400 s
Simulation Area	1000 × 1000
Transmission Range	250 m
Mobility scenario	Random waypoint
Radio Propagation Model	Two-ray ground
MAC Protocol	IEEE 802.11 DCF
MAC Rate	2 Mbps
Confidence interval	95%
Number of nodes	20–50
